# The Hospital

**Published:** 1918-03-23

**Authors:** 


THE
### INSTITUTIONAL WORKER
**Being a Special Supplement to "The Hospital"**

### OUR BUREAU OF INFORMATION.

**Rules for Correspondents.**

1. Every letter must be accompanied by the coupon to be cut from the back cover (inside page) of The Hospital, current issue, and must contain the name and address of the correspondent with pseudonym for publication if desired. Replies by post cannot be given save under exceptional circumstances at the Editor's discretion.

2. Letters from Approved Homes in reply to special needs published in the Bureau should state terms and full particulars, and be sent prepaid under cover to the Editor of the Bureau with name written across coupon for identification.

3. Proprietors of Homes which have not yet been entered on the List of Approved Homes, but have spare accommodation likely to suit special needs, are invited to write for an application form for registration. The fee for registration, which includes two announcements of the Home in the Bureau and other privileges, is 10s.

4. All communications to be addressed to the Editor of The Hospital, 28 Southampton Street, Strand, London, W.C. 2, and marked "Bureau of Information."

### INSTITUTION FACTS AND FIGURES.

### How to Sterilise Rubber Gloves.

**Answer to February Question.**

**By "Margaret."**

The question was :

**Describe the best method of sterilising rubber gloves, to be used during operations, to ensure their being perfectly sterilised with the minimum amount of damage to the rubber.**

Dry Sterilising.

First thoroughly dry and powder the gloves on both sides, leaving them right side out; put them in pairs, both thumbs together, double them, and draw them inside each other. Next put a piece of gauze in the double end to allow the steam to penetrate the glove. Wrap the gloves up in a piece of muslin along with a small amount of powder tied up in a piece of gauze; put in a drum and pack loosely together with a piece of wool between each pair. Place a piece of muslin on he top and sterilise in the ordinary way. I give gloves the same amount of steam as I give all dressings. They never stick together, are comfortable to put on and work in, and they last well even after repeated sterilisation. If sterilised the day before use no powder is necessary to get them on. I have used this method for four years now, and we average 1,900 operations annually. They can be sterilised quite as well in ordinary packets if no drums are available. They always keep a nice colour.

Wet Method.

This is done by boiling for ten minutes, after which they are kept in a large jar of boiled water. With this method the gloves get large after repeated boiling and go a whitish colour. They do not last as long as by the dry method, are not comfortable to wear, and if patched the patches boil off.

### Hot Dinner-Plates.

**Answer to February Question.**

The question was :

**A hospital has four wards, each containing twenty beds. The dinners are served from one inadequate general kitchen and there is no arrangement for heating the plates. Each ward has a very tiny kitchen, which holds a small gas-ring. How can the plates be best heated ?**

**By "M. B. M."**

It is assumed that the dinner plates have to be heated by some means in the very tiny kitchen attached to each ward, except, perhaps, in winter-time, when there may be a fire in the ward where they could be heated.

The maximum number of plates required would be forty, but as it is hardly likely that all the twenty patients would be on full diet this number would not always be in use. However, arrangements for heating forty plates would have to be made in any case.

One method would be to put the required number of plates in a bowl. Take a kettle of *boiling* water, sufficient quantity to cover the plates (they may be done in two lots—*i.e.*, meat and pudding), pour the water over the plates and allow them to stand in it for a few minutes until they get hot, pour the water out of the bowl and dry the plates quickly and use.

While the meat plates are being used the water can be boiling for the pudding plates to be done in the same way. If the water in the taps is hot it should not use a great quantity of gas to boil the kettle. The wardmaid or even a nurse could dry the plates.

As the arrangement must be a permanent one, another method that might be adopted satisfactorily would be to have a bar shelf arranged over the gas-ring—either one that can be moved when not in use—*i.e.*, a tripod arrangement, or if the ring is near a wall a flap shelf or bar of perforated material could be attached to the wall which could be let down out of the way when not in use. This method would probably use more gas than the kettle of boiling water, and would, therefore, be more expensive.

If the wards were on the same floor level or on two floor levels it might be worth while considering an arrangement for the four wards to have the plates heated in one central place—*e.g.*, a small gas-oven might be placed in a convenient spot—as there is gas it would not be a difficult matter, and as the arrangement would be permanent the expense would be justified.

**By "Bluebird."**

I should suggest that the matron or housekeeper make a purchase of four one-gallon saucepans, either of block tin or enamel, and distribute one to each ward kitchen.

Then, about one hour before dinners are sent up from the general kitchen, the ward nurse or junior probationer should light the gas-ring, and put on the pan about three-parts full of water, and, instead of the lid, place as many dinner-plates as will be needed on the saucepan; when the water boils fast, lower the gas; and the plates will get nicely heated all through and keep hot until required.

The water can afterwards be used towards the washing-up of the dinner things.

If the saucepans are well dried after using they should last several years without replacing, so there would only be the initial expense of purchase.

### MARCH QUESTIONS.

### (1) The Admission of Patients.

Is it an advantage to admit patients to small hospitals (under 50 beds) by subscribers' letters, or is the system of admission on a doctor's order only preferable?

### (2) Repairs and Renewals.

Describe the system adopted at your institution for dealing with repairs, including (a) the arrangement in force for the periodical examination of the structure, and (b) the method of collecting, recording, and disposing of repair requisitions.

### RULES.

The following rules must be observed:—

1. Contributions must be written on one side of the paper. Brevity and terseness are desirable features. The MSS. must bear the name and address of the sender and be accompanied by coupon to be cut from the back cover (inside page) of the current issue of THE HOSPITAL. A pseudonym must be chosen if the name is not to be published.
2. Contributions must be addressed to the Editor of THE HOSPITAL, Institutional Worker Supplement, 28 & 29 Southampton Street, Strand, London, W.C. 2, should reach him before the end of the current month, and be marked in the left-hand corner "Facts and Figures."

**A minimum payment of Five Shillings will be made for each published answer.**

### QUESTIONS INVITED.

IN connection with our Question Box, we offer every month five shillings each for the two best questions which are sent in for consideration. The questions may be on any institutional subject and concern any department, from the secretary's to the porter's, or the matron's to the domestic staff; the one essential is that they must be practical and deal with points that have a definite relation to hospital or institutional administration, upkeep, finance, or management. Points relating to artificers' work, housekeeping, the laundry, out-patients, and other practical matters will be welcome. It is hoped that thus, when difficult or doubtful points arise in the course of their work, institutional workers will be encouraged to put them in the form of a question and send them to the Editor, THE HOSPITAL Bureau of Information, 28 & 29 Southampton Street, Strand, W.C. 2, marked "Question Box."

The best questions will be published in due course and our readers will be given the opportunity of answering them. Thus all institutional workers may be able to co-operate with the view of helping the work and smoothing the difficulties of each other. In short, we want the Question Box of the *Institutional Worker* to be freely used by every worker habitually.

### ENQUIRIES AND ANSWERS.

### *WAR MATTERS.*

### Post in Military Hospital.

WE suggest that you answer advertisements which appear in the nursing papers for nurses in military hospitals. You might also write to the Matron-in-Chief, Joint War Committee, 83 Pall Mall, London, S.W. 1, and inquire if your certificate and training qualify you for a position such as you desire.—H. P. B.

### Military Staff Nurse's Post.

FOR a post of staff nurse under the British Red Cross Society you must apply to the Matron-in-Chief, 83 Pall Mall, London, S.W. 1, and the Territorial Force Nursing Service to the Matron-in-Chief, 80 Pall Mall, London, S.W. 1. Give full details of your training and experience when applying to either Matron-in-Chief.—STEWART.

### *THE SICK AND IN NEED.*

### Treatment for Stammering.

WE suggest that you take the advice of your family doctor in this matter. The treatment required must necessarily depend upon the cause of the stammering. If your doctor agrees that it is of nervous origin, possibly one of the hospitals for nervous diseases in London, such as the National Hospital, Queen Square, Bloomsbury, W.C., or the West End Hospital for Diseases of the Nervous System, 73 Welbeck Street, London, W., might give you the necessary treatment, or your doctor might refer you to a specialist.—G. R.

### *THE HOUSEKEEPERS' DEPARTMENT.*

### Food Values.

THE relative values of meat and fish expressed in calories per oz. are: meat, 69; pork, 117; bacon, 169; herring, 30; cod, 14; mackerel, 23; salmon, 41. The meats are mainly composed of protein and fat; fish mainly of protein; a higher percentage of fat is present in salmon, herring, and mackerel than in cod.—C. A. M.

### Tinned Foods.

THERE is no necessity in these days to regard tinned vegetables or fruits, or, in fact, any tinned stuffs, with suspicion. At one time it was found that under certain conditions tin became absorbed to some extent in canned fish. Now, however, owing to greatly improved methods of canning and the rigid system of inspection at the port of entry, the danger of contamination by tin is no doubt reduced to a minimum. All tinned stuffs should, of course, be promptly removed from the tin when opened, and not kept long or taken in excess.—CORA.

### *EMPLOYMENT AND TRAINING.*

### Cottage Nursing Association.

WE suggest that you apply to the Cottage Benefit Nursing Association, Denison House, Vauxhall Bridge Road, London, S.W. 1. Candidates for probationership must be between twenty-three and forty-five years of age, and must be prepared to undergo whatever training is considered advisable. An agreement has to be entered into to serve one of the branches, after training for three and a half years if trained for four months, or four years if trained for six months or longer. A salary of £18 is given during the first and second year, rising £2 annually to £22, with a bonus of £7 7s. if a nurse has served the shorter period or £8 8s. for four years' service. Lodging and uniform are provided.—OLIVER.

### Children's Training in Edinburgh.

THERE is the Royal Hospital for Sick Children, Edinburgh, and we suggest that you write to the matron and inquire if there is a vacancy for a probationer. Five paying probationers are also taken. Ordinary probationers are taken between the ages of nineteen and thirty for three years' training. Paying probationers are taken for six months' training; the fee is £26 5s.—JEAN.

### *MISCELLANEOUS.*

### Steam Boiler Accessories.

YOU can get your steam gauge repaired satisfactorily and expeditiously by J. C. Stewart and Co., of Short Strand Engineering Works, Belfast, who make a speciality of these repairs.—E. T. S.

### Exposure Meter for Radiography.

YOUR trouble is probably due to the fact that your experience is insufficient for the calculation of current exposures. While the efficiency of each set of apparatus varies and the readings given by an exposure meter must consequently be modified in accordance with your own experience of your apparatus, the use of an exposure meter will nevertheless prevent you from making gross errors in your calculations. If you will take the trouble to ascertain the facts required for the proper setting of your meter—*i.e.*, the penetration of your tube (which can be measured by a radiometer), the distance from anode to plate, and the thickness and density of part to be radiographed. A special index is supplied with exposure meters which substituted for the last requirement. Messrs. Watson and Sons, of 196 Great Portland Street, W. 1, supply a useful meter for the sum of £1 1s. only. We advise you to purchase one—it will save the wastage of many exposure plates.—V. A. D.

